# Pasture rewetting in the context of nature conservation shows no long-term impact on endoparasite infections in sheep and cattle

**DOI:** 10.1186/s13071-022-05155-4

**Published:** 2022-01-21

**Authors:** Katharina May, Katharina Raue, Katrin Blazejak, Daniela Jordan, Christina Strube

**Affiliations:** 1 Institute for Parasitology, Centre for Infection Medicine, University of Veterinary Medicine Hannover, Buenteweg 17, 30559 Hannover, Germany; 2 Institute of Animal Breeding and Genetics, Justus-Liebig-University of Gießen, 35390 Gießen, Germany

**Keywords:** *Eimeria* spp., *Fasciola hepatica*, Gastrointestinal nematodes, Grassland, Lungworms, Rumen flukes, Soil drainage

## Abstract

**Background:**

Nature conservation with reduced drainage of pastures has been increasingly promoted in agriculture in recent years. However, moisture on pastures is a crucial factor for the development of free-living stages of many parasite species in ruminants. Hence, for the first time, we conducted a field study between 2015 and 2017 at the German North Sea coast to investigate the long-term effect of pasture rewetting (since 2004) on endoparasite infections in sheep and cattle.

**Methods:**

We examined faecal samples of 474 sheep and 646 cattle from five farms in spring, summer and autumn each year for the presence of endoparasite infections. Animals were kept on conventionally drained, undrained and rewetted pastures. The association between pasture rewetting and endoparasite infection probability was analysed in generalized linear mixed models and including further potential confounders.

**Results:**

Infection frequencies for gastrointestinal strongyles, *Eimeria* spp. and *Strongyloides papillosus* were significantly higher in sheep (62.9%, 31.7% and 16.7%) than in cattle (39.0%, 19.7% and 2.6%). *Fasciola hepatica* was detected with a frequency of 13.3% in sheep and 9.8% in cattle, while rumen fluke frequency was significantly higher in cattle (12.7%) than in sheep (3.8%). *Nematodirus* spp., lungworms (protostrongylids, *Dictyocaulus viviparus*), *Moniezia* spp., *Trichuris* spp. and *Dicrocoelium dendriticum* were identified in less than 7% of samples. Co-infection with more than three endoparasite taxa was present significantly more often in sheep than in cattle. We identified significant positive correlations above 0.2 for excretion intensities between *S. papillosus* with strongyles, *Eimeria* spp. and *Nematodirus* spp. in sheep and between strongyles and *Nematodirus* spp. in cattle. Pasture rewetting had no long-term effect on endoparasite infections, neither in sheep nor in cattle. Interestingly, *F. hepatica* infections decreased significantly in sheep and cattle from 2015 (10.9% and 13.9%) to 2017 (1.4% and 2.1%).

**Conclusions:**

Pasture rewetting for nature conservation did not increase endoparasite infection probability in ruminants in the long term. This finding should be confirmed in ongoing studies aimed at further animal welfare parameters. The rapid decrease in *F. hepatica* infections over 3 years may suggest climatic impact or competition with rumen flukes in addition to potential anthelmintic treatment after feedback of the results to the farmers.

**Graphical Abstract:**

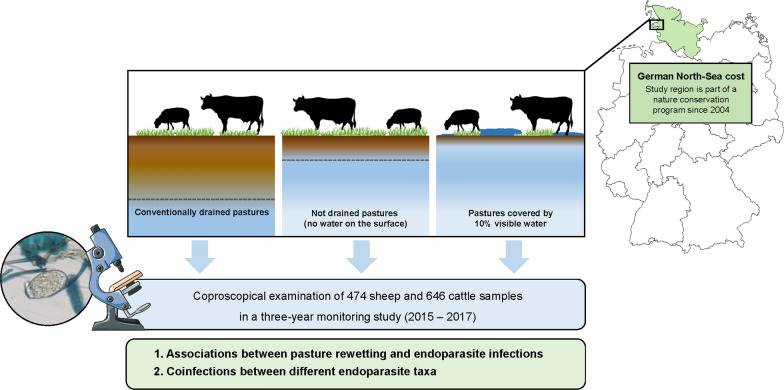

## Background

Endoparasite infections represent a persistent problem in pasture-based farming with a negative impact on animal health and livestock production [1]. Rainfall, moisture, vegetation and grazing management are the main environmental risk factors for helminth and protozoan infection in grassland systems [2–4]. Wet grasslands and moisture are described as crucial factors for the development of environmental, i.e. free-living parasite stages and, in the case of liver and rumen flukes, of their intermediate hosts [4]. Both flukes and snail hosts gain a survival advantage on damp meadows as a consequence of moisture and increased rainfall [5]. Khadijah et al. [6] and Niven et al. [7] identified soil moisture and moisture on pastures as promoting factors for the migration of trichostrongylid larvae onto herbage. In contrast, van Dijk [8] observed that nematode larvae migration was independent of the presence of free water on pastures. In addition to natural climatic fluctuations, human impacts (e.g. pasture soil drainage) may affect endoparasite infection dynamics in grazing ruminants, but are largely unknown to date. In the course of climate protection policy, there has been an increasing trend in recent years towards nature conservation in agriculture [9]. Nature conservation with rewetting of extensively used grasslands and peatlands has the potential to reduce greenhouse gas emissions and to protect genetic diversity [9, 10]. However, rewetting of grasslands may promote endoparasite infections in grazing ruminants due to increased moisture on pastures, so that nature conservation might impair animal welfare and productivity.

Hence, we investigated endoparasite infections in sheep and cattle kept in drained and rewetted pastures on the German North Sea coast peninsula of Eiderstedt. Eiderstedt is located at an altitude of 0–1 m above sea level [11], with a mean temperature of 6.0 °C during the winter season and 14 °C during the summer season [13]. The peninsula’s landscape is characterized by extensive and wet grasslands, representing harsh environmental conditions for sheep and cattle. Grazing pastures are separated by water ditches instead of fences. In 2004, a nature conservation program was initiated in Eiderstedt aimed at traditional landscape usage and promotion of pasture rewetting instead of drainage [12]. In the following years, Kemper and Henze [11] investigated the effect of nature conservation and the impact of rewetting in Eiderstedt on endoparasite infections in cattle during a 3-year monitoring program (2005–2007). The authors identified a negative effect of pasture rewetting on nematode infections in cattle, while they observed no significant effect on *Eimeria* spp. infections.

In the current study, we aimed to follow up the study by Kemper and Henze [11] and to investigate for the first time the long-term effect of rewetting in the course of nature conservation on endoparasite infections in both sheep and cattle. Furthermore, we present the first field study analysing differences in patent co-infections with protozoan and helminth pasture-borne parasites between sheep and cattle kept within the same area.

## Methods

### Study area and animals

The study was conducted during a 3-year period from 2015 to 2017 on the northern German peninsula of Eiderstedt as a follow-up to the study by Kemper and Henze [11]. Eiderstedt is located in the German federal state of Schleswig–Holstein and covers an area of 30,000 ha, with 16,000 ha of extensive grassland which is traditionally used for grazing. According to the German Meteorological Service [13], mean temperatures were 3.0–9.9 °C during the winter season and 10.0–18.0 °C during the summer season between 2015 and 2017. Mean humidity of 82.5% and total rainfall of approximately 980 mm per year were documented in 2015–2017. During the three study years, five farms (one sheep farm, one cattle farm and three farms with both sheep and cattle) were visited each at the beginning of April (spring), July (summer) and November (autumn) to collect faecal samples for coproscopical examinations. Holstein–Friesian and Shorthorn were the main cattle breeds, while the sheep were predominantly East Frisian milk sheep and Texel sheep breeds. Within the framework of the study, animals were kept on grassland pastures during the grazing season from the beginning of April to the end of November according to a nature conservation program. In this program, pastures were assigned to three wetting areas: conventionally drained control pastures (CDPs), non-drained pastures but without water on the surface (NDPs), and pastures 10% covered by visible water (WPs). The distribution of animals within farms and wetting areas was cross-classified, i.e. at least two different wetting areas per farm (despite one cattle farm where cattle had access only to WPs). Rotational grazing of the same animals from one wetting area to another (e.g. from CDPs to NDPs) was not conducted within a year, but between different study years. The animals were kept on pastures day and night, except on the sheep farm, where sheep were kept on pastures for only 8 to 10 h per day. The average stocking rate was 1.5 sheep or 2–3 cattle per hectare. Co-grazing of sheep and cattle on the same pasture was conducted on only one farm. Depending on seasonal conditions and farm management, some animals were kept in stables between April and November. Antiprotozoal drugs were not used by the farmers. Anthelmintic treatment was not routinely documented during the study.

### Faecal sampling and coproscopical examination

A total of 1120 faecal samples were collected, subdivided into 474 samples of sheep and 646 of cattle (266 first-season grazers [FSG] and 380 multiple-season grazers [MSG]). Overall, 836 faecal samples were collected from pastured animals (421 samples of sheep, 415 samples of cattle), while the remaining 284 samples were collected from stabled animals (53 samples of sheep, 231 of cattle). Whenever feasible, samples were collected rectally. In the absence of opportunities to fix grazing animals, samples were taken from freshly excreted faeces, avoiding the collection of soil-touching portions. A detailed overview of the sheep and cattle faecal samples collected in the three wetting areas and study years is presented in Table 1.

**Table 1:** Number of faecal samples collected from cattle and sheep in the three wetting areas during the study years 2015–2017

Wetted area	Study year															
	2015				2016				2017				2015—2017			
	Cattle total	FSG	MSG	Sheep	Cattle total	FSG	MSG	Sheep	Cattle total	FSG	MSG	Sheep	Cattle total	FSG	MSG	Sheep
CDPs	55	26	29	79	31	0	31	67	35	11	24	75	121	37	84	221
NDPs	34	8	26	52	40	25	15	43	54	14	40	74	128	47	81	169
WPs	43	18	25	18	58	35	23	3	65	27	38	10	166	80	86	31
Total	132	52	80	149	129	60	69	113	154	52	102	159	415	164	251	421

*CDPs* conventional drained pastures, *FSG* first-season grazer, *MSG* multiple-season grazer, *NDPs* not drained pastures but without water on the surface, *WPs* pastures 10% covered by visible water

Individual faecal samples were weighed in three portions of 10 g each for different parasitological examinations. To detect lungworm larvae, Baermann funnels were loaded with faeces on the day of sampling and larvae were allowed to migrate approximately 18 h before microscopic examination. The remaining faeces portions were stored at 4 °C. During the following day, faecal samples were analysed by the flotation method using saturated sodium chloride as flotation solution. Additionally, the sedimentation technique was carried out to investigate for trematode eggs. Rumen fluke eggs were isolated from four cattle samples for subsequent molecular species identification by targeting the internal transcribed spacer (ITS)2 region as described previously [14].

Based on the number of lungworm larvae and the different egg morphotypes and oocysts in the faeces, the respective excretion intensity per taxon was determined semiquantitatively as follows: 0 = no excretion; 0–5 = low excretion; 5–10 = medium excretion; 10–20 = high excretion; > 20 = very high excretion.

### Data analyses

The statistical analyses were performed in SAS version 9.4 (SAS Institute; Cary, NC, USA). We used the PROC FREQ procedure in SAS for all descriptive statistics. Differences in infection status and in endoparasite co-infections between sheep and cattle were analysed using Chi-square tests. Correlations between excretion intensities for different taxa within sheep and cattle were analysed using Spearman’s rank correlation tests. Moreover, excretion intensities determined during coproscopical examinations were binary coded as infected (low, medium, high and very high excretion) or uninfected (no excretion) (= infection status) for subsequent model analyses. *P*-values ≤ 0.05 were regarded as significant for all descriptive statistics and all model analyses.

Generalized linear mixed models (SAS GLIMMIX procedure) were applied to study the effect of pasture rewetting (independent variable) and further environmental and management factors on the binary coded infection status “infected” or “uninfected” (dependent variable) in sheep and cattle separately. The infection status for strongyles (strongylid eggs), *F. hepatica* and *Eimeria* spp. was included as dependent variable separately in each model, resulting in six consecutive model runs. The models contained the binomial distribution as the probability distribution and the logit function as link function. The status of the wetting area (CDPs, NDPs, WPs), farm (four farms each for sheep and cattle), season of parasitological examination (spring, summer, autumn) and the sampling year (2015, 2016, 2017) were included as fixed effects in the sheep model. Faecal samples from sheep kept in stables were excluded from the analysis. The same model was implemented in cattle, including the grazing status (FSG or MSG) as an additional fixed effect. The season “spring” was excluded from the cattle model, since all coproscopical samples from cattle in spring were from stabled animals. Fixed effects in the models were tested for significance by stepwise selection. We selected the model with the smallest Akaike information criterion (AIC) in most of the consecutive runs as the final model. For model validation, we checked the normal distribution of residuals by quantile–quantile plots. The significance of fixed effects from the models was tested via *F*-tests (sum of squares type III test statistics = overall *F*-test). We estimated least-squares means (LSMeans) and differences in LSMeans within fixed effects for the presence of strongyles (strongylid eggs), *F. hepatica* and *Eimeria* spp. in sheep and cattle separately.

## Results

### Frequency of endoparasites

**Fig. 1:**
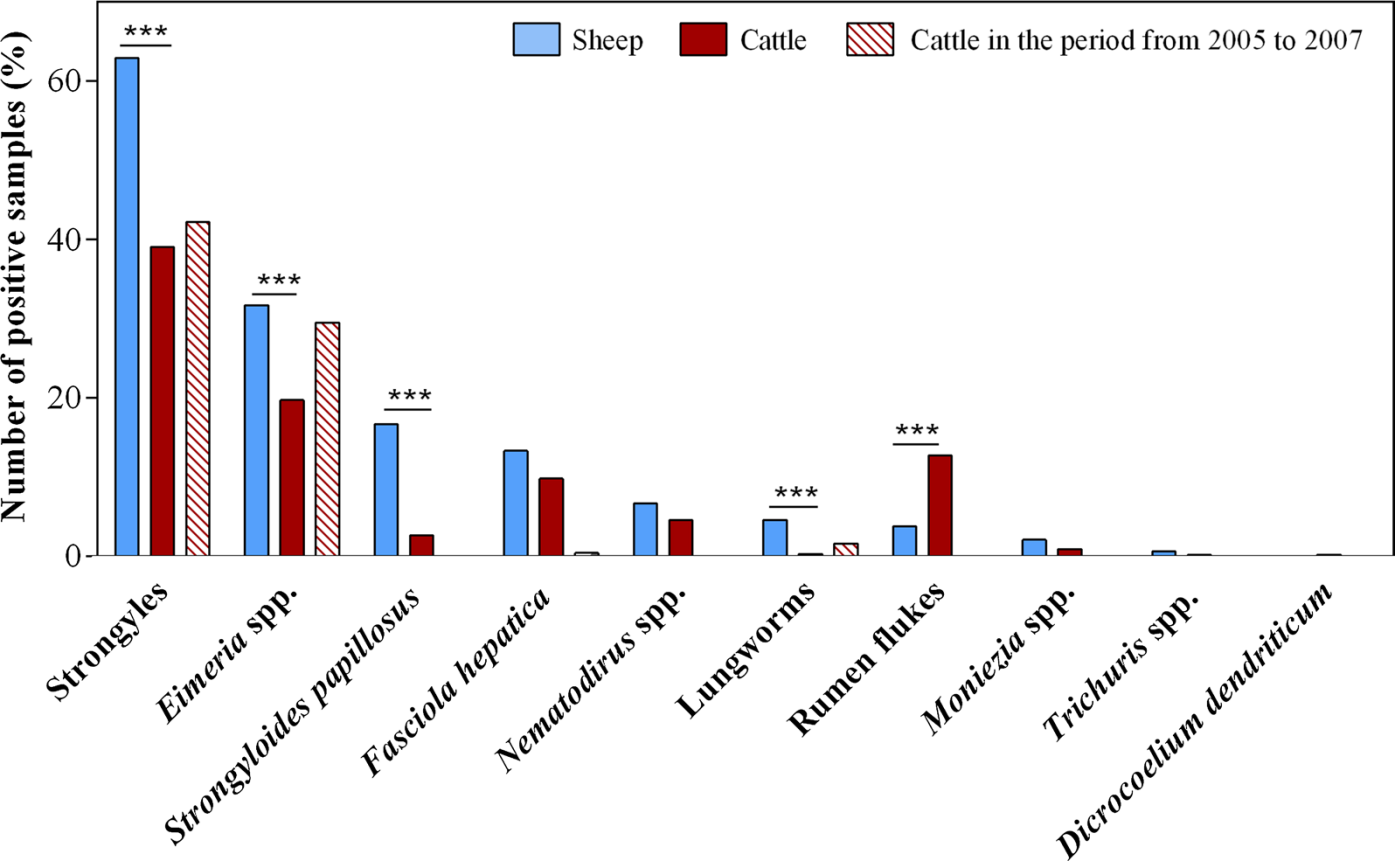
Number of positive sheep and cattle faecal samples (in %) between 2015 and 2017. Additionally, the number of positive cattle samples (in %) between 2005 and 2007 according to Kemper and Henze [11] is shown (note that prevalence was reported for strongyles, *Eimeria* spp., *F. hepatica* and lungworms only). *P ≤ 0.05; **P ≤ 0.001; ***P ≤ 0.0001

An overview of the identified endoparasite taxa in sheep and cattle faecal samples during the study years 2015–2017 is given in Fig. 1. Strongyles (Trichostrongylidae and other Strongylida) were the most frequently detected endoparasite egg type in both sheep and cattle [62.9% (298/474) and 39.0% (252/646) positive samples], followed by *Eimeria* spp. oocysts [31.7% (150/474) and 19.7% (127/646) positive samples]. Compared to sheep, the frequency of strongyle eggs and *Eimeria* oocysts in cattle faeces was significantly lower (Chi-square test; *χ*^2^ = 62.28; *df* = 1;* P* < 0.0001 and *χ*^2^ = 21.10; *df* = 1;* P* < 0.0001). The same applies to *Strongyloides papillosus*, the third most common egg type in sheep with a frequency of 16.7% (79/474) compared to 2.6% (17/646) in cattle samples (Chi-square test; *χ*^2^ = 68.72; *df* = 1;* P* < 0.0001).

Furthermore, lungworm larvae (protostrongylids in sheep, *Dictyocaulus viviparus* in cattle) were diagnosed significantly more often in sheep [4.6% (22/474)] than in cattle samples [0.3% (2/646)] (Chi-square test; *χ*^2^ = 24.46; *df* = 1; *P* < 0.0001), while rumen fluke eggs occurred significantly less often in sheep [3.8% (18/474)] than in cattle samples [12.7% (82/646)] (Chi-square test; *χ*^2^ = 26.61; *df* = 1; *P* < 0.0001). Exemplary molecular rumen fluke species identification in four cattle samples revealed 100% identity with *Calicophoron daubneyi* (GenBank Accession No. KP201674).

In both ruminant species, eggs of *F. hepatica* were detected fourth most often, with a frequency of 13.3% (63/474) and 9.8% (63/646) in sheep and cattle samples, respectively (Chi-square test; *χ*^2^ = 3.43; *df* = 1;* P* = 0.064), followed by *Nematodirus* spp. [6.7% (33/474) and 4.6% (30/646); Chi-square test; *χ*^2^ = 2.77; *df* = 1;* P* = 0.096] and *Moniezia* spp. [2.1% (10/474) and 0.9% (6/646); Chi-square test; *χ*^2^ = 2.71; *df* = 1; *P* = 0.099]. At less than 1%, the frequency of *Trichuris* spp. eggs was low in both ruminants [0.6% (3/474) in sheep and 0.2% (1/646) in cattle samples; Chi-square test; *χ*^2^ = 1.76; *df* = 1;* P* = 0.185], while eggs of *Dicrocoelium dendriticum* were detected in cattle samples only [0.2% (1/646); Chi-square test; *χ*^2^ = 0.73; *df* = 1;* P* = 0.392].

### Endoparasite co-infections and correlations of excretion intensities

**Fig. 2:**
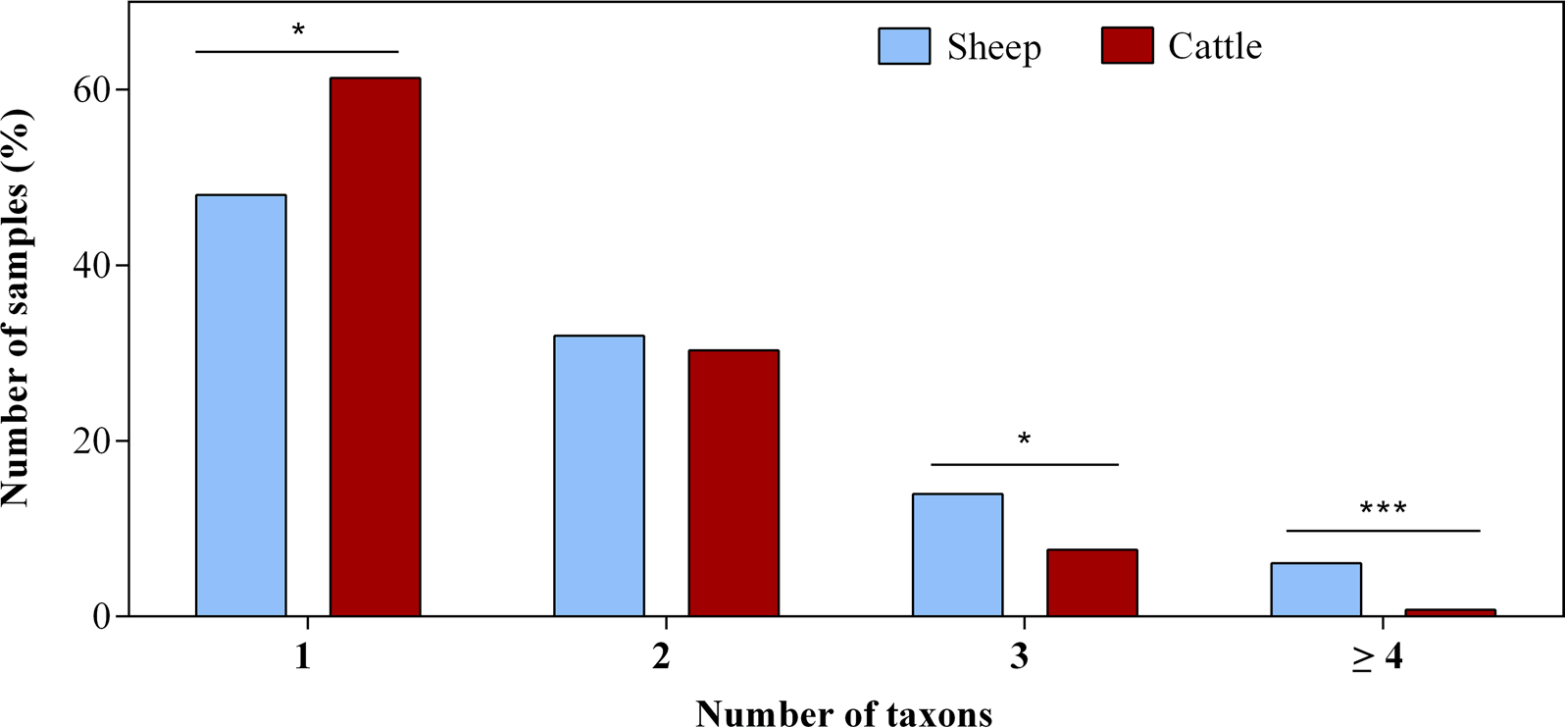
Number of sheep and cattle faecal samples (in %) with one, two, three or ≥ four endoparasite taxa. *P ≤ 0.05; **P ≤ 0.001; ***P ≤ 0.0001

Of the 379 positive sheep faecal samples, 48.0% (182/379) presented one endoparasite type, followed by 31.9% (121/379) with two, 14.0% (53/379) with three, and 6.1% (23/379) with four or more endoparasite taxa (Fig. 2). In cattle, one taxon was identified in 61.3% (241/393) of the positive samples, two in 30.3% (119/393), three in 7.6% (30/393), and four or more taxa in only 0.8% (3/393) of positive samples (Fig. 2). Cattle were mono-infected significantly more often than sheep (Chi-square test; *χ*^2^ = 13.78; df = 1;* P* = 0.0002). Co-infections were observed in 40.1% (152/379) of positive samples in sheep and in 50.1% (197/393) of positive samples in cattle, with no significant difference between host species for total co-infections (Chi-square test; *χ*^2^ = 1.14; df = 1;* P* = 0.2860). However, the number of samples representing co-infection with three or more endoparasite taxa was significantly higher in sheep than in cattle (Chi-square test; *χ*^2^ = 8.11; df = 1;* P* = 0.0044 and Chi-square test; *χ*^2^ = 16.69; df = 1; *P* < 0.0001, respectively).

To test for correlations in excretion intensities, Spearman’s rank correlation tests were applied for all possible pairwise endoparasite combinations (except of *D. dendriticum*) (Table 2). In both sheep and cattle, the excretion intensity of strongyle eggs was significantly positively correlated with that of *Eimeria* spp. (sheep: *rs* = 0.097; *P* = 0.035; cattle: *rs* = 0.173; *P* < 0.0001), *S. papillosus* (sheep: *rs* = 0.212; *P* < 0.0001; cattle: *rs* = 0.093; *P* = 0.018) and *Nematodirus* spp. (sheep: *rs* = 0.095; *P* = 0.039; cattle: *rs* = 0.283; *P* < 0.0001). Furthermore, a significant positive correlation between *Eimeria* spp. and *Nematodirus* spp. excretion intensity was observed in both ruminants (sheep: *rs* = 0.161; *P* = 0.0004; cattle: *rs* = 0.121; *P* = 0.002), while only sheep showed significant positive correlations of *S. papillosus* with *Eimeria* spp. (*rs* = 0.249; *P* < 0.0001) and *Nematodirus* spp. (*rs* = 0.248; *P* < 0.0001). In cattle, *Nematodirus* spp. excretion intensity was significantly positively correlated with that of *Trichuris* spp. (*rs* = 0.175; *P* < 0.0001) and lungworms (*rs* = 0.123; *P* = 0.002). For the latter, a significant positive correlation with *S. papillosus* was found in sheep (*rs* = 0.151; *P* = 0.001). Moreover, Spearman’s rank correlation test revealed that in sheep, increased excretion of *F. hepatica* eggs was associated with increased excretion of rumen fluke eggs (*rs* = 0.092; *P* = 0.046) and *Trichuris* spp. eggs (*rs* = 0.137; *P* = 0.003). No significant negative correlations in excretion intensities were found in either ruminant species.

**Table 2:** Spearman’s rank correlation tests (*rs*) of endoparasite excretion intensities in sheep (*n* = 474; below diagonal) and cattle (*n* = 646; above diagonal)

	Strongyles	*Eimeria* spp.	*S. papillosus*	*F. hepatica*	*Nematodirus* spp.	Lungworms	Rumen flukes	*Moniezia* spp.	*Trichuris* spp.
Strongyles	–	0.173***	0.093*	−0.027	0.283***	0.008	−0.038	0.045	0.043
*Eimeria* spp.	0.097*	–	0.035	−0.042	0.121*	−0.027	−0.035	−0.048	0.092*
*S. papillosus*	0.212***	0.249***	–	−0.019	0.056	−0.009	−0.063	−0.016	−0.006
*F. hepatica*	0.075	−0.071	−0.014	–	−0.049	−0.018	0.051	0.021	−0.013
*Nematodirus* spp.	0.095*	0.161**	0.248***	−0.083	–	0.123*	−0.064	−0.021	0.175***
Lungworms	−0.002	0.038	0.151*	−0.086	0.019	–	0.055	−0.005	−0.002
Rumen flukes	0.023	−0.021	−0.059	0.092*	−0.012	−0.044	–	−0.037	−0.015
*Moniezia* spp.	0.042	0.010	0.016	0.072	−0.040	−0.032	−0.029	–	−0.004
*Trichuris* spp.	0.035	−0.004	−0.036	0.137*	−0.022	−0.018	−0.016	−0.012	–

Significant correlations are printed in bold

*P ≤ 0.05, **P ≤ 0.001, ***P ≤ 0.0001

### Impact of rewetting on endoparasite infections

Model results for the test of significance for fixed effects (overall *F*-test) in sheep and cattle are given in Tables 3 and 4, respectively. The overall *F*-test showed that the wetting area had no significant effect on endoparasite infections in either sheep or cattle (*P* > 0.05).

**Table 3:** Results of the overall *F*-test (*P*-values) of fixed effects on endoparasite infections in sheep

Fixed effect	Strongyles	*Eimeria* spp.	*Fasciola hepatica*
Wetted area	0.1499	0.0845	0.8252
Farm	**0.0006**	0.1619	**0.0003**
Season	** < 0.0001**	**0.0063**	0.5514
Sampling year	0.8112	0.7631	** < 0.0001**

Significant *P*-values are printed in bold

**Table 4:** Results of the overall *F* test (*P*-values) of fixed effects on endoparasite infections in cattle

Fixed effect	Strongyles	*Eimeria* spp.	*Fasciola hepatica*
Wetted area	0.7844	0.1618	0.1429
Farm	0.1981	0.1335	0.2128
Season	0.1826	0.9813	0.1131
Sampling year	0.2359	** < 0.0001**	** < 0.0001**
Grazing period	** < 0.0001**	**0.0007**	**0.0009**

Significant *P*-values are printed in bold

**Fig. 3:**
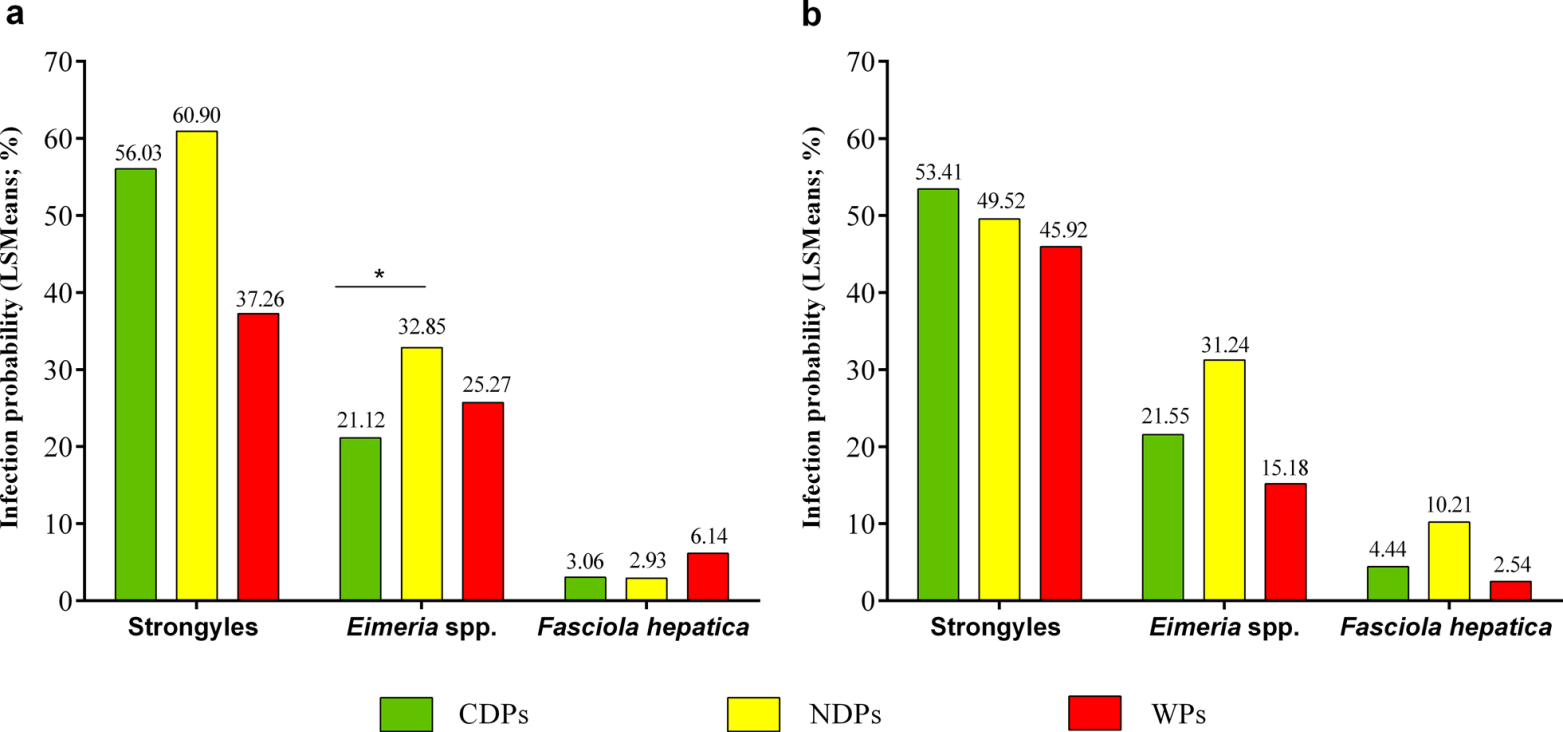
Least-squares means (LSMeans) for gastrointestinal strongyle, *Fasciola hepatica* and *Eimeria* spp. infections within rewetting areas (CDPs, NDPs, WPs) for **a** sheep and **b** cattle. *P ≤ 0.05; **P ≤ 0.001; ***P ≤ 0.0001

In sheep, LSMeans revealed the highest infection probability for gastrointestinal strongyles and for *Eimeria* spp. for NDPs (60.90% and 32.85%, respectively) (Fig. 3; Table 5). For *Eimeria* spp., the difference in LSMeans observed for NDPs (32.85%) compared to CDPs (21.12%) was statistically significant (*P* ≤ 0.05) (Fig. 3 and Table 5). Regarding *F. hepatica*, infection probability was highest in WPs (6.14%; *P* > 0.05).

**Table 5:** Least-squares means (LSMeans) with corresponding standard error (± SE) for the infection probability with gastrointestinal strongyles, *Eimeria* spp. and *F. hepatica* in sheep and cattle within fixed effect classes included in the model

Fixed effects	Effect class	Sheep			Cattle		
		Strongyles	*Eimeria* spp.	*F. hepatica*	Strongyles	*Eimeria* spp.	*F. hepatica*
Wetting area	CDPs	56.03 ± 0.05^a^	21.12 ± 0.04^a^	03.06 ± 0.02^a^	53.41 ± 0.06^a^	21.55 ± 0.05^a^	04.44 ± 0.02^a^
	NDPs	60.90 ± 0.04^a^	32.85 ± 0.04^b^	02.93 ± 0.01^a^	49.52 ± 0.06^a^	31.24 ± 0.06^a^	10.21 ± 0.04^a^
	WPs	37.26 ± 0.11^a^	25.72 ± 0.09^a,b^	06.14 ± 0.07^a^	45.92 ± 0.08^a^	15.18 ± 0.06^a^	02.54 ± 0.03^a^
Farm	1	56.90 ± 0.06^a,d^	33.07 ± 0.05^a,c^	24.54 ± 0.09^a^	–	–	–
	2	28.22 ± 0.08^b^	13.94 ± 0.06^b^	02.67 ± 0.03^b^	38.20 ± 0.07^a,b^	22.80 ± 0.06^a,c^	01.91 ± 0.01^a,b^
	3	74.31 ± 0.05^a,c^	31.71 ± 0.05^b,c^	02.28 ± 0.02^b^	50.52 ± 0.05^b,c^	14.77 ± 0.04^a,b^	03.46 ± 0.02^b,c^
	4	–	–	–	52.96 ± 0.07^b,c^	21.75 ± 0.08^a,c^	21.04 ± 13.61^b,c^
	5	45.33 ± 0.08^b,d^	30.33 ± 0.06^b,c^	01.17 ± 0.01^b^	56.96 ± 0.07^c^	30.66 ± 0.07^c^	03.68 ± 0.02^c^
Season	Spring^1^	40.31 ± 0.06^a^	18.37 ± 0.04^a^	04.34 ± 0.02^a^	–	–	–
	Summer	45.08 ± 0.05^a^	36.00 ± 0.05^b^	02.99 ± 0.02^a^	45.84 ± 0.04^a^	21.92 ± 0.03^a^	06.49 ± 0.02^a^
	Autumn	68.01 ± 0.05^b^	26.39 ± 0.05^a,b^	04.27 ± 0.02^a^	53.39 ± 0.04^a^	22.03 ± 0.03^a^	03.70 ± 0.01^a^
Sampling year	2015	53.14 ± 0.06^a^	24.96 ± 0.04^a^	10.92 ± 0.04^a^	51.07 ± 0.05^a^	34.91 ± 0.04^a^	13.88 ± 0.03^a^
	2016	48.84 ± 0.07^a^	25.48 ± 0.05^a^	03.54 ± 0.02^b^	54.41 ± 0.05^a^	31.53 ± 0.05^a^	03.92 ± 0.02^b^
	2017	52.13 ± 0.05^a^	28.52 ± 0.05^a^	01.37 ± 0.01^c^	43.39 ± 0.05^a^	08.30 ± 0.02^b^	02.05 ± 0.01^b,c^
Grazing period	FSG	–	–	–	66.78 ± 0.04^a^	31.23 ± 0.04^a^	02.35 ± 0.01^a^
	MSG	–	–	–	32.53 ± 0.03^b^	14.87 ± 0.02^b^	09.99 ± 0.02^b^

*CDPs* conventional drained pastures, *FSG* first-season grazers, *MSG* multiple-season grazers, *NDPs* not drained pastures but without water on the surface, *WPs*  pastures 10% covered by visible water

^1^The season “spring” was excluded from the cattle model, since all coproscopical samples from cattle in spring were from stabled animals

Different superscripts (a–d) within fixed effect classes and columns indicate significant differences (*P* ≤ 0.05)

In cattle, LSMeans for *Eimeria* spp. and *F. hepatica* infections were highest for NDPs (31.24% and 10.21%, respectively; *P* > 0.05), while CDPs posed the highest infection probability for gastrointestinal strongyles (53.41%; *P* > 0.05) (Fig. 3; Table 5).

### Impact of season on endoparasite infections

The season significantly influenced the infection probability for gastrointestinal strongyles (*P* < 0.0001) and *Eimeria* spp. (*P* = 0.0063) in sheep according to the results from the overall *F*-test (Table 3). We estimated significantly higher LSMeans for strongyle infections in autumn (68.01%) than in summer (45.08%) or spring (40.31%) (Table 5). For *Eimeria* spp., infection probability was significantly higher in summer (36.0%) than in spring (18.37%) (Table 5). The season had no significant effect on *F. hepatica* infections in sheep (Table 3).

In cattle, the overall *F*-test revealed no significant effect of season on the three tested endoparasite infections, and no significant differences in LSMeans between summer and autumn were identified (*P* > 0.05; Table 4).

### Impact of sampling year on endoparasite infections

**Fig. 4:**
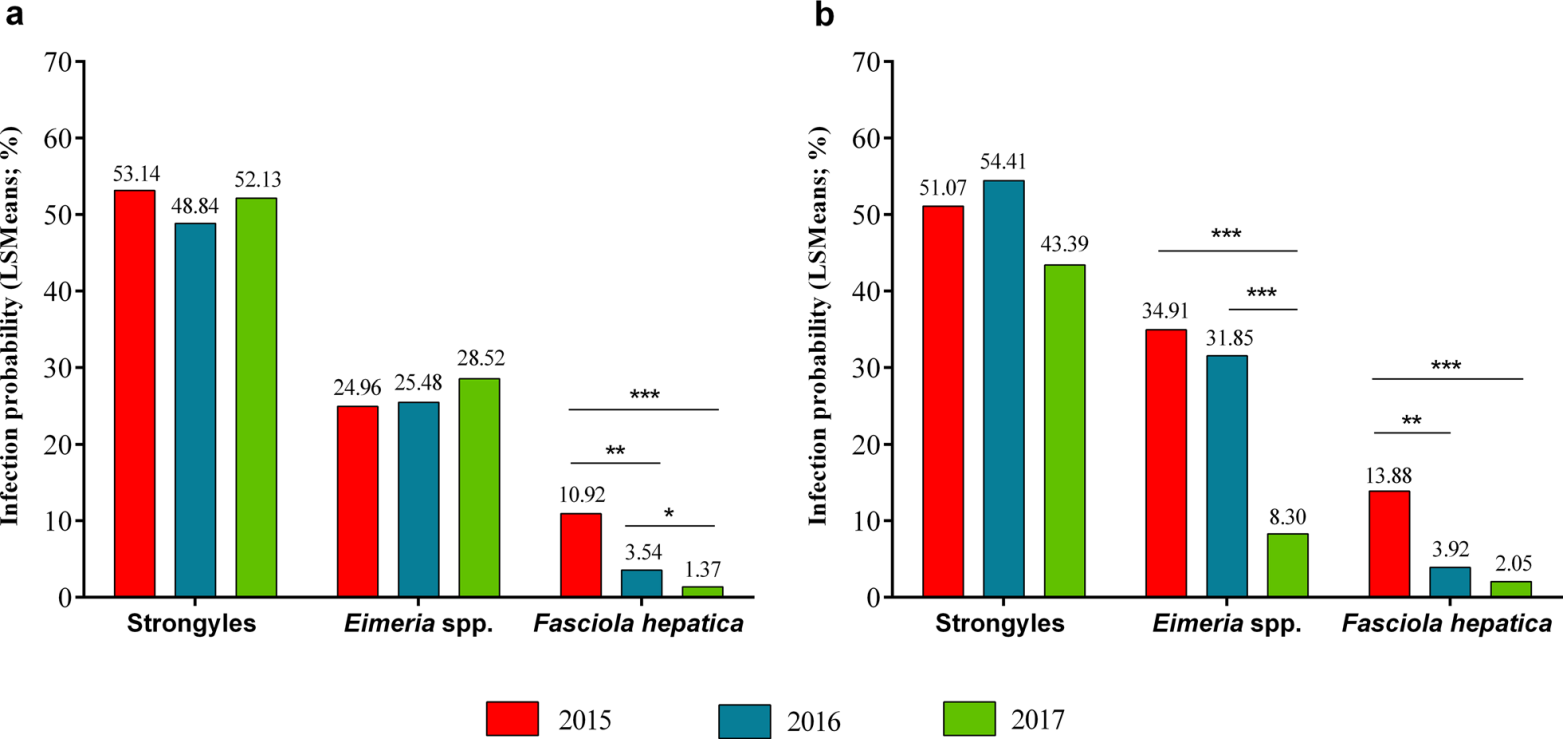
Least-squares means (LSMeans) for gastrointestinal strongyle, *Fasciola hepatica* and *Eimeria* spp. infections within sampling years (2015–2017) for **a** sheep and **b** cattle. *P ≤ 0.05; **P ≤ 0.001; ***P ≤ 0.0001

The overall *F*-test showed that the sampling year significantly influenced *F. hepatica* infections in sheep (*P* < 0.0001), while gastrointestinal strongyle and *Eimeria* spp. infections were unaffected (*P* > 0.05) (Table 3). In sheep, we estimated significantly higher LSMeans for *F. hepatica* infections in 2015 (10.92%) than in 2016 (3.54%; *P* = 0.002) and 2017 (1.37%; *P* < 0.0001) (Fig. 4; Table 5). Moreover, *F. hepatica* infection probability in sheep was significantly higher in 2016 than in 2017 (*P* = 0.05; Fig. 4; Table 5).

In cattle, the overall *F*-test revealed a significant effect of sampling year on *Eimeria* spp. and *F. hepatica* infections (*P* < 0.0001; Table 4), but not on gastrointestinal strongyle infections (*P* = 0.2359). LSMeans for *Eimeria* spp. infections in cattle were significantly higher in 2015 and 2016 (34.91% and 31.85%; *P* < 0.0001) than in 2017 (8.30%) (Fig. 4; Table 5). Regarding *F. hepatica* infections in cattle, infection probability decreased significantly from 2015 (13.88%) to 2016 (3.92%; *P* = 0.0019) and 2017 (2.05%; *P* < 0.0001) (Fig. 4; Table 5).

### Impact of grazing period on endoparasite infections

The grazing period included in the models for cattle (FSG vs. MSG) significantly influenced all three endoparasite infections according to the overall *F*-test (*P* < 0.001; Table 4). LSMeans for gastrointestinal strongyle and *Eimeria* spp. infections were significantly higher for FSG (66.78% and 31.23%, respectively) than for MSG (32.53% and 14.87%, respectively) (*P* < 0.0001 and *P* = 0.0007, respectively; Table 5), while MSG had a significantly higher infection probability for *F. hepatica* infections (9.99%) than FSG (2.35%) (*P* = 0.0009; Table 5).

## Discussion

Climate change, fluctuations in precipitation rates and soil moisture are known to affect the life-cycle and the development of many parasite species in ruminants [15, 16]. Moisture is known to prevent egg desiccation and death of developing strongyle larvae [4, 17, 18], and additionally, larval motility can be inhibited on dry pastures [17]. Moreover, the dependence of *F. hepatica* and rumen fluke free-living stages and their intermediate snail host on sufficient moisture has been vividly shown [4, 15]. In the present study, we analysed the long-term effect of different soil moisture gradients (drained vs. rewetted pastures) on the most frequent endoparasite infections in sheep and cattle in the context of a nature conservation program established in 2004 in the German federal state of Schleswig Holstein [12]. In both ruminant species, gastrointestinal strongyles (hereinafter referred to only as strongyles) were most frequently detected, followed by *Eimeria* species. Additionally, *F. hepatica* infections were analysed in detail as it can be assumed that these increase due to rewetting. However, based on the overall *F*-test statistic results from the model analyses, no long-term effect of pasture rewetting on strongyle, *Eimeria* spp., or *F. hepatica* infections were identified, neither in sheep nor in cattle. This finding is in contrast to the results by Kemper and Henze [11], who identified a significant influence of pasture rewetting on strongyle infections including 692 cattle samples from six farms in the same study area in a similar model analysis. The 3-year monitoring study by Kemper and Henze [11] was conducted in 2005–2007, and four of the six farms also participated in our study. In the previous study, the strongyle infection probability was significantly lower in CDPs (19%, referred to as “green areas” in Kemper and Henze [11]) than in NDPs (55%, “yellow areas”) or WPs (37%, “red areas”). This difference might have become relative in the time leading up to our study, for example due to changes in anthelmintic treatment strategies over the years. However, it should also be noted that Kemper and Henze [11] only examined a relatively small amount of faeces in the flotation method (2 g compared to 10 g in our analyses), which could have led to samples with only a low number of eggs being classified as negative.

For *F. hepatica*, the highest (but not significantly different) infection probabilities were estimated for sheep on WPs and cattle on NDPs. Machatschek et al. [19] observed an increased liver fluke prevalence after the destruction of drainage systems on alpine pastures. Hence, we expected significantly lower prevalence for *F. hepatica* infection in animals kept in CDPs, but observed no significant effects, suggesting that these pastures are also moist enough to efficiently maintain *F. hepatica* infection cycles in the study area. Interestingly, we observed a significant decrease in *F. hepatica* infections in sheep and cattle, with estimated frequency of almost 11% and 14%, respectively, in 2015 to values lower than 2% in 2017. In contrast, we detected eggs of rumen flukes in 12.7% of all faecal samples from cattle, with a rapid increase in frequency from 6.2% in 2015 to 25.7% in 2017. A possible explanation might be an increased use of anthelmintics effective for *F. hepatica* but not for rumen flukes after feedback of the study results in 2015 to the farmers. The same observation was made by Jones et al. [20] in sheep and cattle in the United Kingdom. The authors hypothesized that treating against *F. hepatica* reduces the number of *F. hepatica* eggs shed onto pastures, and thus may potentially increase *G. truncatula* infections with rumen flukes. An increase in rumen fluke infections was also observed in other Western European countries, showing that paramphistomosis is an emerging disease in ruminant livestock populations [21–24], with *C. daubneyi* as the predominant rumen fluke species in both cattle and sheep in Europe, including Germany [14, 21, 25]. Climate change factors, the availability of a suitable snail intermediate host, and the movement of livestock favoured the introduction and increasing prevalence of *C. daubneyi* in Western Europe [26]. Abrous et al. [27] and Naranjo-Lucena et al. [25] suggested that the *F. hepatica* cercariae shedding pattern from snails is more sensitive to variations in temperature than that of *C. daubneyi*, which is better adapted to daily temperature changes. Notably, we identified a significant, fourfold higher rumen fluke frequency in cattle than in sheep. Under the assumption that both species were similarly exposed, sheep might be more resistant to rumen fluke infections than cattle. This has been confirmed in other studies evaluating both natural [25, 28, 29] and experimental infections [30].

Another important parasite in grazing cattle, the bovine lungworm *D. viviparus*, was identified in only 0.3% of the samples, which is considerably lower than the frequency of 1.59% in the previous study by Kemper and Henze [11]. In individual cows originating from 17 north-western German farms, patent infections were identified in 0.9% and 3.4% of cows in summer and autumn 2015, respectively [31]. The decreased lungworm frequency in the area investigated in the present study and the rather low infection rates observed by May et al. [31] raise the question of whether *D. viviparus* is declining in (northern) Germany. This assumption is supported by a recent seroepidemiological study investigating bulk tank milk (BTM) samples from northern, eastern and southern Germany during 2017–2019 [32]. In total, only 2.3% of the BTM samples tested were positive; in northern Germany the percentage amounted to 4.5%. The authors note that these rates are remarkably lower than those in a study using the same enzyme-linked immunosorbent assay (ELISA) about 10 years earlier (in 2008), where the BTM seroprevalence for the included German regions was determined at 10.4–31.2% [33]. More precisely, seroprevalence in northern Germany in 2008 was 17.6–22.3%, of which 17.6% was in the federal state of Schleswig–Holstein, where our study area, the peninsula Eiderstedt, is located. As also suggested by Springer et al. [32], further studies are needed to clarify whether the cattle lungworm is really on the decline in Germany.

A significant decrease in *Eimeria* spp. frequency from 2015 (34.91%) and 2016 (31.53%) to 2017 (8.30%) was estimated in cattle, while the frequency in sheep did not differ significantly among the three study years. Season had a significant effect on *Eimeria* spp. and strongyle infections in sheep, while cattle infections were unaffected by seasonal variations. Kemper and Henze [11] detected a frequency of 62% strongyle infections in cattle in autumn, while in summer and spring only 40% and 27% of samples revealed positive results. The same pattern was observed in our study for the sheep. Hamer et al. [34] observed a well-established pattern of ovine gastrointestinal nematode infections with a spring periparturient rise in ewes on three sheep farms in the UK, while infections and egg counts in lambs peaked in autumn.

Remarkably, *S. papillosus* was the third most common endoparasite in sheep, detected in 16.7% of the faecal samples and significantly more frequently than in the cattle samples (2.6%). Balicka-Ramsz et al. [35] detected a similar proportion of 18% positive sheep in southern Poland during summer. For lambs and calves, prevalence of up to 60% was observed [36, 37], while prevalence in older animals amounted to only 20% [38, 39]. Of the *S. papillosus*-positive cattle samples in our study, 58.8% were from FSG and 41.2% from MSG (data not shown). Similarly, the grazing period significantly influenced strongyle, *Eimeria* spp. and *F. hepatica* infections. As expected, strongyle and *Eimeria* spp. frequencies were significantly higher in FSG due to the build-up of immunity in older cattle [40, 41], while the *F. hepatica* frequency was significantly higher in MSG.

When analysing gastrointestinal co-infections, we identified significant positive low to moderate correlations between individual excretion intensities of strongyle eggs and *Eimeria* spp. oocysts, *S. papillosus* and *Nematodirus* spp. eggs in both sheep and cattle. According to our findings, Craig et al. [42] identified a significant moderate positive correlation between strongyles and *Nematodirus* spp. in lambs and between strongyles and coccidia in yearlings and adult sheep. An explanation was given by Gorsich et al. [43], who suggested that suppression of host immune response by one parasite may increase the likelihood or severity of infection with another parasite. Interestingly, we found no significant correlation between strongyles and *F. hepatica* in either sheep or cattle. Co-infection with strongyles and *F. hepatica* was detected in only 10.1% of sheep and 3.6% of cattle samples (data not shown). In contrast, Bellet et al. [44] identified 39% of cattle co-infected with *Ostertagia* spp. and liver flukes by post-mortem examination of 974 cattle. On the herd level, a recent study identified 14.9% and 22.4% of BTM samples in northern and southern Germany, respectively, with co-exposure to *O. ostertagi* and *F. hepatica* [32], and a Swiss BTM serosurvey detected positive correlations between these parasites in multiple regression models [45]. However, antibody-based co-infection rates are less reliable than coproscopical ones due to the persistence of antibodies beyond the infection, which may falsely elevate co-infection rates.

Spearman’s rank correlation between *F. hepatica* and rumen fluke egg excretion was weakly positive but significant in our study in sheep (0.09, *P* ≤ 0.05), but not in cattle (0.05, *P* ≥ 0.05). Jones et al. [20] found co-infection with *F. hepatica* and *C. daubneyi* in 46% of sheep and cattle farms in Wales, United Kingdom, and a significant negative correlation (−0.36) between the parasite infection intensities within the farms. This discrepancy relative to our results arouses interest for further studies on co-infection with *F. hepatica* and rumen flukes and their mutual influence in sheep and cattle.

## Conclusions

The results of the present study indicate that endoparasite infections in ruminants are not affected by pasture rewetting in the long term. Therefore, the nature conservation program on the peninsula of Eiderstedt has no lasting negative impact on animal health and welfare with regard to endoparasite infections. Further studies are needed to investigate whether our results can be extrapolated to other regions or similar conservation programmes. Regarding epidemiological parameters, we found co-infection with more than three endoparasite taxa significantly more often in sheep than in cattle. Significant positive but no significant negative correlations of excretion intensities between different parasite taxa were found in both ruminant species. Interestingly, the bovine lungworm *D. viviparus* was detected in only 0.3% of all cattle samples, and *F. hepatica* infections decreased significantly from 2015 to 2017 in both sheep and cattle, while at the same time the rumen fluke frequency increased in cattle. Future studies are warranted to follow up on these epidemiological findings.

## Data Availability

Data supporting the findings of this study are available within the article.

## Abbreviations

BTM: Bulk tank milk
CDPs: Conventionally drained pastures
FSG: First-season grazers
LSMeans: Least-squares means
MSG: Multiple-season grazers
NDPs: Not drained pastures, but without water on the surface
WPs: Pastures 10% covered by visible water
